# Education Competencies for Integrative Oncology—Results of a Systematic Review and an International and Interprofessional Consensus Procedure

**DOI:** 10.1007/s13187-020-01829-8

**Published:** 2020-08-11

**Authors:** Claudia M. Witt, Lynda G. Balneaves, Linda E. Carlson, Misha Cohen, Gary Deng, Judith M. Fouladbakhsh, Anita Y. Kinney, Ashwin Mehta, Josh Mailman, Laura Pole, Alizé A. Rogge, Carole O’Toole, Suzanna M. Zick, Stefanie M. Helmer

**Affiliations:** 1 Institute for Complementary and Integrative Medicine, University Hospital Zurich and University of Zurich, Sonneggstrasse 6, 8091 Zurich, Switzerland; 2 Institute for Social Medicine, Epidemiology, and Health Economics, Charité – Universitätsmedizin Berlin, corporate member of Freie Universität Berlin, Humboldt-Universität zu Berlin, and Berlin Institute of Health, 13353 Berlin, Germany; 3 College of Nursing, Rady Faculty of Health Sciences, University of Manitoba, Winnipeg, Canada; 4 Department of Oncology, Cumming School of Medicine, University of Calgary, Calgary, Canada; 5 Chicken Soup Chinese Medicine, San Francisco, CA USA; 6 American College of Traditional Chinese Medicine at California Institute of Integral Studies, San Francisco, CA USA; 7 Memorial Sloan Kettering Cancer Center, New York, NY USA; 8 Oakland University, School of Nursing, Rochester, MI USA; 9 Department of Epidemiology, School of Public Health and Rutgers, Cancer Institute of New Jersey, New Brunswick, New Jersey USA; 10 Memorial Health Care System, FL, Hollywood, USA; 11 NorCal CarciNET Community, Oakland, CA USA; 12 Smith Center for Healing and the Arts, Institute for Integrative Oncology Navigation, Washington, DC USA; 13 Smith Center for Healing and the Arts, Washington, DC USA; 14 Department of Family Medicine and Nutritional Sciences Schools of Medicine and Public Health, University of Michigan, Ann Arbor, Michigan USA

**Keywords:** Cancer, Core competencies, Integrative oncology, Interprofessional collaboration, Consensus procedure

## Abstract

Integrative oncology is a burgeoning field and typically provided by a multiprofessional team. To ensure cancer patients receive effective, appropriate, and safe care, health professionals providing integrative cancer care should have a certain set of competencies. The aim of this project was to define core competencies for different health professions involved in integrative oncology. The project consisted of two phases. A systematic literature review on published competencies was performed, and the results informed an international and interprofessional consensus procedure. The second phase consisted of three rounds of consensus procedure and included 28 experts representing 7 different professions (medical doctors, psychologists, nurses, naturopathic doctors, traditional Chinese medicine practitioners, yoga practitioners, patient navigators) as well as patient advocates, public health experts, and members of the Society for Integrative Oncology. A total of 40 integrative medicine competencies were identified in the literature review. These were further complemented by 18 core oncology competencies. The final round of the consensus procedure yielded 37 core competencies in the following categories: knowledge (*n* = 11), skills (*n* = 17), and abilities (*n* = 9). There was an agreement that these competencies are relevant for all participating professions. The integrative oncology core competencies combine both fundamental oncology knowledge and integrative medicine competencies that are necessary to provide effective and safe integrative oncology care for cancer patients. They can be used as a starting point for developing profession-specific learning objectives and to establish integrative oncology education and training programs to meet the needs of cancer patients and health professionals.

## Introduction

The use of complementary and integrative medicine (CIM) by cancer patients [1] and cancer survivors [2] is widespread with meta-analytic evidence showing that more than 40% of cancer patients use CIM [3]. The term “integrative oncology” has been defined [4] using a consensus process, by the Society for Integrative Oncology (SIO [5]), as “a patient-centered, evidence-informed field of cancer care that utilizes mind and body practices, natural products, and/or lifestyle modifications from different traditions alongside conventional cancer treatments. Integrative oncology aims to optimize health, quality of life, and clinical outcomes across the cancer care continuum and to empower people to prevent cancer and become active participants before, during, and beyond cancer treatment” [6].

Founded in 2003, SIO is an interprofessional non-profit organization whose mission is to advanced evidence-based, comprehensive integrative healthcare to improve the lives of people affected by cancer. Through education, research, and knowledge transfer initiatives, such as an annual international conference and the development of clinical practice guidelines, SIO’s vision is to have research inform the integration of complementary modalities into oncology care so that evidence-based integrative oncology care is accessible and standard for all patients across the cancer continuum. SIO provides much needed, evidence-informed leadership and collaborative opportunities to the interdisciplinary integrative oncology research and clinical practice communities around the world.

As integrative oncology involves various healthcare professionals [7], its implementation into clinical practice requires a divergent set of competencies [8]. Although integrative oncology content exists in courses, curricula, syllabi, and trainings, information about the required core competencies is incomplete and not yet standardized [8, 9]. To date, no core set of education competencies for integrative oncology that reflects different professions and countries has been developed. This may be due to heterogeneous education systems and activities across countries as well as different legal, ethical, regulatory, and political influences on the practice of integrative oncology. As such, the primary aim of this project was to systematically develop a set of core education competencies for integrative oncology that would be applicable to a wide range of healthcare providers from different educational backgrounds and countries.

## Methods

The absence of clearly defined core competencies for integrative oncology was seen by the SIO Board at its retreat in 2016 as a quality-related problem in integrative healthcare. To close the gap between what is and what is desired, a model that has been used by the Institute for Health Care Improvement in the USA called the “Collaborative Model for Achieving Breakthrough Improvement” has been adapted to the needs of the project [10]. After the topic of the core competencies was identified, the SIO Board members representing different professions were recruited for the project. The project consisted of two phases, and the teams for the phases were selected based on their expertise. Phase I included a systematic review of the literature, the identification of relevant competencies, as well as categorizing them. In Phase II, building on the results of the systematic review, an international and interprofessional consensus procedure was conducted to develop a set of core competencies for healthcare professions who deliver integrative oncology care.

### Systematic Literature Review

#### Literature Inclusion and Exclusion Criteria

Publications focused on education integrative oncology competencies for physicians, nurses, integrative oncology practitioners, and other healthcare professionals that were published in scientific journals or as reports, consensus papers, and working papers or theses were included. Publications were excluded if the education competencies or activities did not include CIM in the context of cancer, if the reporting of the education activity did not include competencies or details about the curricula, or if the publication was not available in English or German.

#### Search Strategy

Scientific literature about integrative oncology education and information about education activities, such as curricula, syllabi, and course objectives, were searched and analyzed to get an overview about required core competencies. The search strategy was conducted using an explicit and reproducible methodology in the following electronic databases from inception until February 6, 2017: Ovid MEDLINE, CENTRAL, CINAHL, EMBASE, PsychINFO, PsychARTICLES, and Web of Science. The search included all types of papers published related to competencies in integrative oncology by using the following keywords or free text words in combination with subject headings, where available: disciplines possibly related to integrative oncology (integrative oncology OR complementary medicine OR alternative medicine OR integrative care OR integrative nursing OR integrative medicine) AND educational element (course OR curriculum OR education or program* OR session or teaching OR training OR workshop OR competencies OR value OR knowledge OR attitude OR skill* OR mission* OR vision* OR syllab*) AND type of publication (evaluation OR investigation OR study or trial OR proposal OR examination OR research OR survey). MeSH terms were used to restrict the results to literature specific to oncology, depending on the respective database. In addition, the SIO members were asked to provide gray and/or unpublished literature on education competencies.

#### Selection of Studies

All items identified by the literature search were entered into a bibliographic database. One reviewer thoroughly checked all searched items by assessing titles and abstracts, excluding clearly ineligible articles based on the search criteria and aim of the project. Full text copies were obtained of all remaining articles and assessed by two reviewers independently for eligibility. Publications were excluded only with the agreement of both reviewers. Reasons for exclusion were documented and any disagreements resolved by discussion. If several publications for a single study were published, all publications were reviewed if they met the eligibility criteria.

#### Data Extraction

Two reviewers extracted data from selected publications using a standardized form. The results of the data extraction were collated into the categories, “knowledge,” “skills,” and “abilities” (KSA), to structure the results and to summarize findings. The KSA classification was drawn from the basic core competency model used by the Association of American Medical Colleges for Entering Medical Students [11]. In this classification, knowledge was defined as a body of information applied directly to the performance of a function; skills as observable competence to perform a learned psychomotor act; and ability as a competence to perform an observable behavior or a behavior that results in an observable product. Any uncertainties regarding data extraction and classification for specific publications were discussed by the reviewers, with disagreements resolved by consensus and the final decisions resolved by a third reviewer.

### Consensus Procedure

In Phase II, a consensus procedure was performed involving a task force, an extended expert group, and knowledgeable SIO members. The initial set of competencies developed from the literature review and additional core oncology competencies was further refined through a multistep process that was guided by the task force. The task force consisted of 12 experts representing seven different professions (medical doctors (MDs), psychologists, nurses, naturopathic doctors (NDs), traditional Chinese medicine (TCM) practitioners, yoga practitioners, patient navigators) and additional relevant perspectives (public health and patient advocates). Each task force member identified further experts from a similar professional background representing three different regional areas (Asia/Australia, Europe, North America). The extended expert group helped broaden the expertise represented in the consensus procedure and allowed perspectives from different international regions to be included. The consensus process involved three online surveys and an onsite survey at an international integrative oncology conference (SIO conference in 2017) as well as direct feedback from the task force members. Each survey was followed by a task force conference call and emails to revise the competencies.

In the first online survey, the importance of each competency found in the literature was rated by the task force and extended experts on a scale from 0 (not important) to 10 (very important). In addition, the task force indicated their agreement with the placement of the competencies in one of the KSA categories, and new core competencies that were not listed could be suggested.

The second online survey was again forwarded to the task force and extended experts for review. The revised list of competencies was then prioritized based on how important each competency was for a given healthcare profession (low, moderate, high priority, or not applicable for my profession). The final set of competencies was included in a survey that was sent to all SIO members using a link in a newsletter and was also provided as a hardcopy version at the annual SIO conference in November 2017. Respondents were asked to prioritize and comment on the competencies with respect to the importance for their respective profession. During each conference call, the task force consented agreed upon a threshold that needed to be met to keep a competency on the list. Competencies reaching the threshold were only discussed if at least one task force member wanted to discuss optional changes. All competencies not reaching the threshold were discussed in detail. All decisions during the conference calls were based on full consensus among the group.

## Results

### Systematic Literature Review

**Fig. 1:**
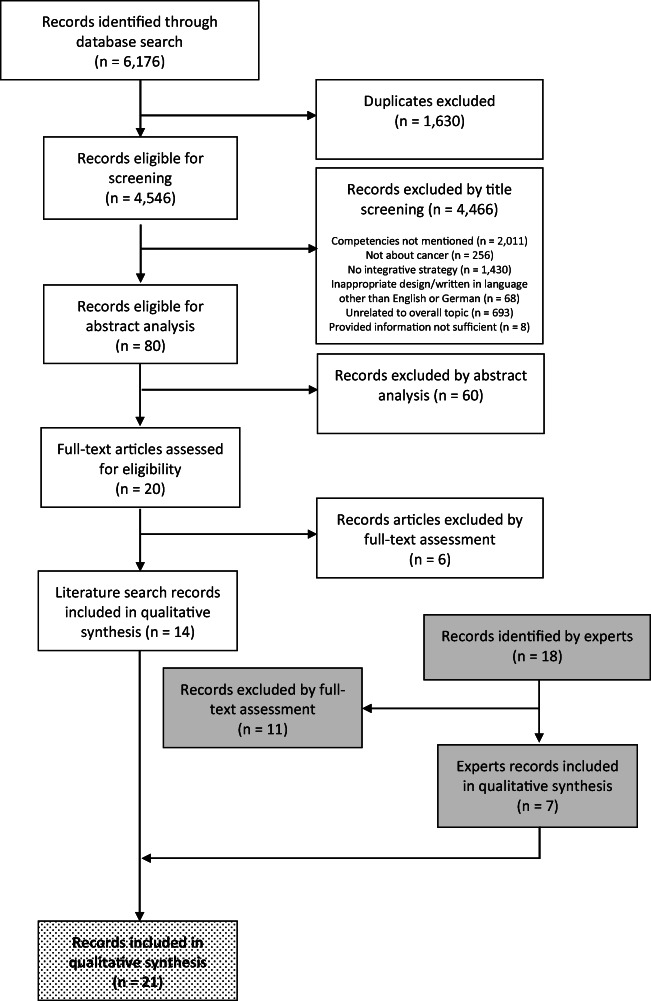
Flowchart for literature search and study selection

The literature review yielded 21 eligible studies (see Fig. 1). The vast majority were from North America (*n* = 10 from the USA, *n* = 2 from Canada) and Europe (*n* = 7). Most publications (*n* = 18) mentioned competencies that could be classified into the three KSA categories. Nearly all publications (*n* = 19) reported competencies for the broader field of integrative medicine, but 2 publications [12, 13] defined competencies that were of special interest for health professionals working in the field of breast cancer oncology. Most publications addressed several professions; 5 publications focused on nurses only [13–17], 3 publications on physicians [18–20], and 1 on medical students [21].

A total of 28 competencies were identified from the literature review, summarized and classified into the KSA categories (see Table 1). Some of these 28 competencies had elaborate descriptions that had to be summarized, and several of the competencies needed to be broken up into single competencies. Following this process, a total of 40 single competencies were developed. In addition, 18 other competencies that are known to be core competencies for medical oncology were added by one task force member (GD) to the list [22]. This resulted in a total of 58 competencies as a starting point for the consensus procedure.

**Table 1:** Overview of competency categories identified in the systematic literature review

Knowledge	Skill	Ability
General knowledge about evidence-based medicine [7, 17, 20, 21, 24–26]	Provide evidence-based, balanced, resource-oriented, up-to-date complementary and integrative medicine (CIM) information that assists patients to make a decision [7, 9, 15, 16, 18–20, 27–31]	Respect individual differences in the understanding and implementation of integrative oncology [17, 19–21, 24, 26]
Knowledge on how to access and appraise scientific literature on integrative oncology [19, 20, 31]	Identify, understand, and contextualize relevant information on CIM [7, 12, 15–19, 21]	Respect cultural and ethnic differences in the understanding and implementation of integrative oncology [7, 21, 29]
Knowledge about cancer [18, 26, 28]	Understand patients, the problems patients face, and their needs [14, 19, 29, 31, 32]	Appreciate a whole person and patient-centered approach [16, 25]
Knowledge about common complementary medicine (CM) therapies, including their history, theory, proposed mechanisms, safety/efficacy profile, contraindications, prevalence, and patterns of use [7, 12–15, 17–20, 26, 27, 29, 31–33]	Engage with patients (and caregivers) to build resilience and resources to best empower patients during cancer treatments [12, 18, 19, 21, 26, 28–30]	Be empathic [12, 19, 29]
Knowledge about services/providers’ quality assurance and reimbursement [19, 21]	Master the principles and practices of communication [12, 18, 19, 21, 26, 28–30]	Respect of patient’s beliefs [7, 12, 17–20]
Knowledge about the principles of a healing environment [14, 17, 21]	Inquire about patients’ use of CIM and their motives [7, 12, 14, 15, 17, 19, 20, 29]	Be open-minded [7, 12, 18–20, 28, 29]
Knowledge about communication theories and strategies [9, 28]	Work in an interprofessional team [14–16, 19, 21, 24, 28]	Be attentive [19]
Knowledge about conventional medical language [15, 24, 30]	Have an adequate training in one or more CIM modalities and be able to apply it to cancer patients [14, 17, 19, 26, 28, 30, 34]	Be self-aware [15, 19, 20, 24, 29, 31]
	Identify suitable CIM providers for a respective patient [15, 16, 19, 20, 25, 29]	Be able to accept that CIM use is often based on no/unclear evidence [29]
	Adequate documentation of interventions and patients’ response to them [14, 15, 21, 24, 29]	
	Use adequate medical terminology [15, 29]	

### Consensus Procedure

A total of 25 experts from 7 different healthcare professions and 3 international regions completed the initial web-based survey (24% from Asia/Australia, 20% from Europe, 56% from North America).

The majority (*n* = 20, 80%) of the participants agreed with the suggested KSA competency categories. The task force decided that all competencies with a level of importance rating specified by median and/or mean of least 9 on the 0–10 (10 being a very important competency) scale would remain on the list. All other competencies were discussed in detail and either rephrased, merged, or deleted. This process led to a total of 38 competencies: 10 knowledge, 15 skill, and 7 ability competencies and an additional 2 knowledge, 2 skill, and 2 ability competencies that were newly developed or extensively modified by the task force.

In the second online survey, 28 experts from 7 different healthcare professions (14% from Asia/Australia, 18% from Europe, 68% from North America) prioritized the competencies according to the importance for their profession and gave feedback on the new or modified competencies. As all competencies met the overall threshold of importance (rated at least moderate or high priority, using the categories low/moderate/high), the task force decided to examine the feedback from the different professions and to retain all competencies that were of high priority for at least 80% of the participants of each profession. All other competencies were again discussed by the task force group and either rephrased (*n* = 1) or omitted (*n* = 1). The final list with 37 competencies included 11 knowledge, 17 skill, and 9 ability competencies.

There was a full agreement among task force members that all 37 competencies were relevant for all participating healthcare professions.

A total of 57 SIO members answered the online or the hard copy survey ((40% MD, 14% TCM specialist, 9% nurses/nurse practitioners) and 36 others (i.e., researcher, students, administrator, yoga practitioners, patient navigators)) and overall agreed about the relevance of the core competency set as shown in their ratings. The findings of the second survey (task force and extended expert group) and third survey (SIO members) are summarized in Table 2.

**Table 2:** Results of the extended expert group and SIO member surveys

Knowledge competencies	Rated as priority (%)		Skill competencies	Rated as priority (%)		Ability competencies	Rated as priority (%)	
Health professionals working in integrative oncology should	Experts	SIO members	Health professionals working in integrative oncology should	Experts	SIO members	Health professionals working in integrative oncology should	Experts	SIO members
Have general knowledge about evidence-based medicine	96.4	100	Provide evidence-based and balanced CM information	96.7	100	Respect individual, cultural, and ethnic differences in the understanding and implementation of integrative oncology	96.4	100
Know how to access and appraise the scientific literature on complementary medicine (CM)	100	96.5	Stay up-to-date with CM information	100	98.2	Appreciate a patient-centered, whole person approach	100	100
Demonstrate the understanding of the basics of history, theory, and mechanisms of common CM therapies	96.4	93.0	Provide reputable websites and other information or resources on CM	100	100	Be empathic, non-judgmental, open minded attentive, and self-aware and respect patients’ beliefs	100	100
demonstrate the understanding of safety/effectiveness, interaction profiles, and contraindications of common CM therapies	100	98.2	Assist patients to make a decision	92.9	93	Establish rapport and form a therapeutic partnership with patient	100	100
Understand the major cancer treatment modalities (surgery, chemotherapy, radiotherapy, endocrine, and biological therapy)	100	91.2	Identify, understand, and contextualize relevant information on CM	96.4	98.2	Identify one’s own knowledge deficiency and know where to find help	100	98.2
List common symptoms associated with cancer	100	94.7	Master the principles and practices of communication, which means an empathic, open, trustful communication that follows common recommendations of communication with cancer patients	100	96.5	Pursue lifelong learning and continuous self-improvement	100	96.5
List common side effects of cancer treatment	100	94.7	Engage with patients (and caregivers) to build resilience and resources to best empower patients during cancer treatment	100	96.5	Respect the strengths and limitations of applying evidence-based medicine principles to the circumstances of an individual patient	100	100
Describe the cancer care continuum	100	94.7	Inquire about patients’ use of CM and their motives	100	93.0	Be able to obtain key information regarding the patient’s cancer history: type of cancer, types of previous treatments (surgery, chemotherapy, radiation, endocrine, targeted therapy), current disease stage, and current treatment	92.9	92.9
Discuss the psycho-social-cultural context of cancer care	100	96.5	Work in an interprofessional team	100	96.5	Help patient understand the risks and benefits of evidence-based CM approaches so that they may choose care that aligns with their values and goals	96.4	98.2
Discuss the distinction between the terms “healing” and “curing”	96.4	96.5	Understand patients, the problems patients face, and their needs	100	98.2			
Have knowledge and or ability to obtain information about services/providers’ quality assurance, licensing government regulation, and reimbursement of CM	100	91.2	Identify CM providers for a patient	89.3	94.7			
			adequately document interventions and patients’ response to them	100	94.7			
			Use appropriate medical terminology	96.4	94.7			
			Assess patients’ psycho-social-cultural environment and identify barriers to proper care	100	95.7			
			Implement a personal self-care strategy (may include nutrition awareness, self-regulatory techniques, exercise, journaling, creative arts, spirituality, mind body skills, etc.)	100	96.5			
			Discuss CIM in the context of different types of cancer	92.8	93.0			
			Be able to obtain information about cancer pathogenesis, the general course of the disease, and treatment outcomes of common cancers	92.8	93.0			

Percentage of respondents that rated competencies as moderate or high priority for their profession

## Discussion

This study is among the first to identify core competencies for integrative oncology healthcare providers. Based on an iterative process including a comprehensive literature review by an expert task force and multi-disciplinary oncology providers and by a survey of members of SIO, a final set of 37 core competencies for integrative oncology was identified. These 37 competencies were further categorized into knowledge, skills, and abilities and agreed on by representatives from seven different professions from Asia, Europe, and North America.

Searching the literature on integrative oncology competencies and complementing it with current fundamental knowledge in oncology will ensure that future healthcare providers who develop these competencies are competent and able to take a safe and knowledgeable approach to integrative oncology care. The consensus procedure incorporated practical experiences and perspectives from different professions and international regions to be embedded in the competencies. In addition, we partially utilized a model that has been used by the Institute for Health Care Improvement for breakthrough advancement in health care [10]. However, we adapted this model to our needs. The so-called action periods that typically take place between the “learning sessions” (exchange between experts) were used for discussions within each profession because implementing the changes (competencies) and measuring the outcomes as typically done within this model would have taken too long for the scope of this project. An alternative result of the project would have been developing different core competencies for each healthcare profession, which would have been supported by the general approach called for in the Collaborative Model for Achieving Breakthrough Improvement. Interestingly, there was full consensus on having the same competencies for all professions, which will make it much easier to inform about the results and measure the impact in the future.

Validation by an even broader international group was also possible by giving SIO members the opportunity to provide feedback on the competency list. However, the study also had limitations. We only included papers in English and German in the systematic review. In addition, surveys typically do not reach all stakeholders, and only those SIO members who have a strong educational interest might have completed the member survey. In addition, SIO members might reflect a unique group of healthcare providers in integrative oncology, resulting in a response bias. They might be more drawn to a scientific, evidence-based, and interprofessional approach and see fundamental oncology knowledge as a basis for integrative oncology. Nevertheless, a strong advantage of SIO is that it is an interprofessional organization with integrative oncology experts from world-leading cancers centers. As such, integrative oncology as represented by this set of core competencies would reflect an approach that can be integrated in cancer centers globally and aims for best outcomes and to provide best care.

The development of a core set of competencies for integrative oncology that encompasses seven professions highlights the interprofessional nature of the field and the potential for future development of interprofessional trainings to benefit cancer patients and improve outcomes.

The 37 core competencies defined in this study are an important starting point and inform future integrative oncology education and training programs for different healthcare professions.

It is important to distinguish between the different sectors of health care when applying education competencies for integrative oncology. In acute care situations, such as brief hospital stays (e.g., fever during chemotherapy) or emergency room visits, an integrative oncology approach will play a less important role. In contrast, in outpatient care situations (e.g., ambulatory chemotherapy or radiation therapy), lengthier hospital stays, or during palliative care, it is of higher importance given the potential role of integrative oncology therapies. For those professions who are part of the cancer care team and are engaged in the integrative oncology care, the core competencies will play a more substantive role. Nevertheless, each profession will have to determine which of the defined integrative oncology competencies are already part of their undergraduate curricula (e.g., MD or nursing degrees) and which will need to be embedded in graduate and continuing education courses and programs. Furthermore, profession-specific and perhaps even country-specific competencies may require development and detailed learning objectives, and didactical approaches would have to be defined.

Competencies are of high relevance because the evidence for selected CIM interventions is growing. If patients decide, based on the advice of their oncology healthcare provider, to pursue a CIM treatment, it would be of limited help if the provider does not have relevant core competencies for integrative cancer care [23]. This core set of integrative oncology competencies will help to have more competent providers in the future, who provide evidence-based care for symptom reduction and quality of life improvement of cancer patients and are able to avoid negative aspects of those interventions such as time herb-drug interactions.

## Data Availability

The raw data supporting the conclusion of this manuscript will be made available by the corresponding author on reasonable request.
